# Rift Valley Fever Outbreak, Mayotte, France, 2018–2019

**DOI:** 10.3201/eid2604.191147

**Published:** 2020-04

**Authors:** Hassani Youssouf, Marion Subiros, Genevieve Dennetiere, Louis Collet, Laure Dommergues, Amandine Pauvert, Patrick Rabarison, Christelle Vauloup-Fellous, Gilles Le Godais, Marie-Christine Jaffar-Bandjee, Maxime Jean, Marie-Claire Paty, Harold Noel, Sophie Oliver, Laurent Filleul, Christine Larsen

**Affiliations:** Santé Publique France, Mayotte, France (H. Youssouf, M. Subiros, C. Larsen);; Agence Régionale de Santé Océan Indien, Mayotte (G. Dennetiere, P. Rabarison);; Centre Hospitalier de Mayotte, Mayotte (L. Collet, C. Vauloup-Fellous, M. Jean, S. Olivier);; Cooperative Agricole des Eleveurs Mahorais Coconi, Mayotte (L. Dommergues);; Direction de l’Alimentation, de l’Agriculture, et de la Foret de Mayotte, Mayotte (A. Pauvert, G. Le Godais);; Université Paris-Sud, INSERM U1193, Villejuif, France (G. Le Godais);; CNR associé des arboviruses, CHU Réunion, CH Félix Guyon, Saint-Denis, La Réunion, France (M. Jaffar-Bandjee);; Santé Publique France, Mayotte, France (M.-C. Paty, H. Noel, S. Olivier, L. Filleul)

**Keywords:** Rift Valley fever, Rift Valley fever virus, epidemiological surveillance, virologic surveillance, outbreak, Mayotte, France, zoonoses, vector-borne infections, viruses

## Abstract

From November 2018 through July 2019, an outbreak of Rift Valley fever in humans occurred in Mayotte, France; 142 cases were confirmed. Exposure to animals or their biological fluid was reported by 73% of patients. Health authorities have been implementing control measures, including veterinary surveys, vector control interventions, and prevention measures.

The southwestern islands of the Indian Ocean are threatened by arbovirus outbreaks because of their tropical climate, geographic proximity to arbovirus-endemic countries, tourism, and numerous commercial exchanges. One such island is Mayotte, an overseas department of France, located between the eastern coast of Africa and Madagascar. The island is densely populated, with ≈280,000 inhabitants at 690 inhabitants/km^2^ (*1**–**3*).

Rift Valley fever (RVF) is a mosquito-borne zoonosis that affects domestic animals and humans. Humans are infected by RVF virus (RVFV) through contact with blood or organs of infected animals, slaughtering or handling infected animals, consuming contaminated meat that was not adequately aged or properly cooked, or consuming raw milk. The virus can also be transmitted through the bite of infected mosquitoes (mainly *Aedes* spp*.* and *Culex* spp.) (*4**,**5*).

In Mayotte, epidemiologic surveillance for arbovirus infections among humans was implemented in 2008. This surveillance is based on using real-time reverse transcription PCR (RT-PCR) to test all patients with suspected dengue-like syndrome for dengue, chikungunya, and Rift Valley fever viruses (*6*) and for *Leptospira* spp. (*7*). Each positive case of RVF is reported by the Centre Hospitalier de Mayotte laboratory to the regional health authority (Agence Régionale de Santé Océan Indien) for implementation of control measures (veterinary investigations and vector control). Every confirmed case is investigated, and clinical and environmental data, including information about exposures, are collected.

In Mayotte, epidemiologic surveillance for RVF in livestock was also implemented in 2008 (*8*). A cross-sectional seroprevalence survey of asymptomatic livestock is conducted yearly. At least 350 samples have been tested each year with a commercial ELISA kit (*9*). For correctly identified cattle, sex, breed, and date of birth are available.

RVFV in humans was detected for the first time in Mayotte in 2007 (*10**,**11*). The genomic analysis of the Mayotte isolates placed them within the 2006–2007 eastern African Kenya-1 lineage (*12*), suggesting importation from mainland Africa. Retrospective analyses of livestock serum (collected from 2004 through 2008) showed that RVFV had been in Mayotte since 2004; however, no sequencing was performed at that time (*13*). In addition, a study conducted in 2011 estimated RVFV seroprevalence in the general human population ≥5 years of age to be 3.5% (95% CI 2.6%–4.8%) (*10*).

## The Study

The first case of RVF in Mayotte was diagnosed by RT-PCR on November 22, 2018, for a patient living in Mamoudzou. Two weeks later, 4 new cases were diagnosed. No case-patient had traveled during the 2 weeks before symptom onset, and the cases were defined as autochthonous. During the same period, analyses conducted by the agricultural cooperative of Mayotte and confirmed by the Centre for International Cooperation in Agronomic Research for Development showed that RVF seroprevalence among cattle had increased from 3.6% (95% CI 2.3%–5.6%) in July 2017–June 2018 to 10.1% (95% CI 6.5%–15.3%) in July–September 2018.

From November 22, 2018, through July 31, 2019, RT-PCR at the Centre Hospitalier de Mayotte confirmed 142 cases of RVF in humans. The epidemic curve peaked in week 7, when 18 cases were confirmed. The last confirmed case of RVF was in week 28 (Figure).

**Figure:**
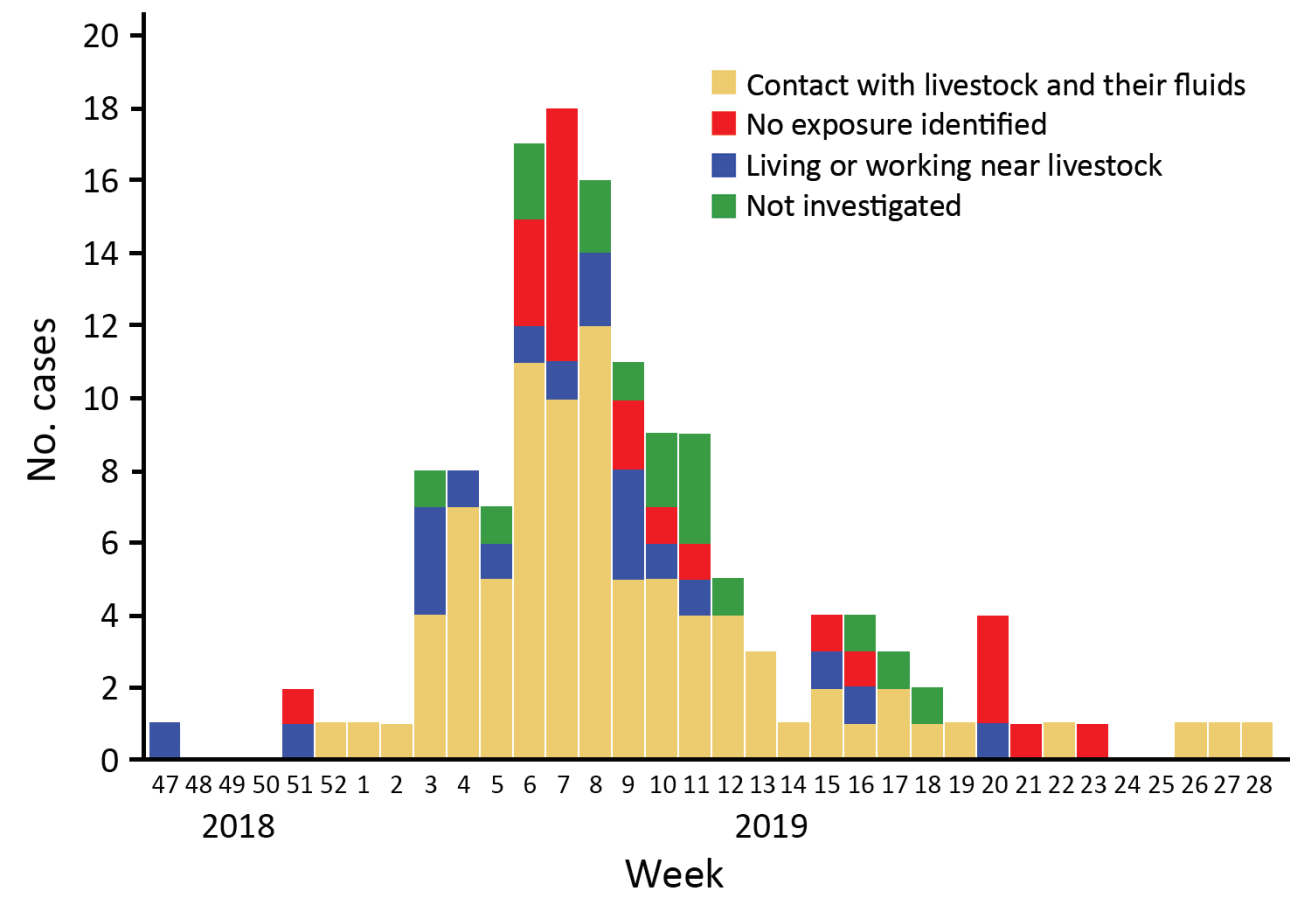
Sources of exposure for 142 patients with Rift Valley fever, by week of laboratory request, in Mayotte, France, 2018–2019.

Most case-patients were male (3 male:1 female), and median age was 41 years (range 4–75 years). Clusters of cases in humans and animals were located mainly in the central and western areas of the island.

Among the 142 human cases, 126 (88.7%) were investigated; of these, 67.5% of case-patients reported having direct contact with livestock (care, treatment, slaughter) or their biological fluids (including consumption of raw or curdled milk), 15.1% reported living or working near livestock, and 22 (17.4%) reported having none of these exposures. Multiple possible exposures (having direct contact with livestock or their biological fluids and living or working near livestock) were declared by 50 case-patients. However, all case-patients had a connection with the affected areas (living, working, or walking in the central or western part of the island), and 4 lived in environments favorable for mosquitoes and mosquito breeding. Furthermore, most case-patients did not use any type of mosquito control in their houses.

Mosquitoes captured by the Agence Régionale de Santé Océan Indien were predominantly of the genus *Culex*. These mosquitoes were not tested for RVFV. In 100 herds of cattle and 26 herds of small ruminants, PCR detected 165 animals (141 cattle, 17 goats, and 7 sheep) positive for RVFV.

Information about clinical signs and symptoms was available for only 97 human case-patients (Table). Two case-patients had meningitis, and 1 was positive for RVFV by RT-PCR of cerebrospinal fluid. This patient had a stiff neck a week after the onset of signs and exhibited the neurologic sign of initial loss of consciousness. Twelve case-patients, including a pregnant woman in the sixth month of gestation, required hospitalization for at least 48 hours.

**Table:** Potential Rift Valley fever virus exposures and clinical signs for persons with Rift Valley fever, Mayotte, France, November 22–July 31, 2019

Variable	No. (%) cases
Risk exposure, n = 142	
Investigated	126 (88.7)
Direct contact with animals and their fluids, including milk	85 (67.5)
Living or working near livestock	19 (15.1)
No contact with animals or consumption of products at risk	22 (17.4)
Not investigated	16 (11.3)
Signs and symptoms reported by patients, n = 97	
Fever	87 (89.7)
Arthralgia	61 (62.9)
Myalgia	42 (43.3)
Retroorbital pain	18 (18.6)
Headache	72 (74.2)
Weakness	49 (50.5)
Nausea/vomiting	31 (31.9)
Cough	5 (5.1)
Other signs and symptoms reported by doctors, n = 142	
Meningitis	2 (1.4)
Meningoencephalitis	1 (0.7)
Meningeal syndrome	3 (2.1)
Neurologic signs	2 (1.4)
Ocular complication	1 (0.7)

Doctors reported 2 severe cases. The first, with a complication of meningoencephalitis, occurred 3 weeks after the first confirmed case. This case-patient was hospitalized for gait disorder, dizziness, confusion, and rapid onset of hemiplegia; the date of symptom onset was March 18. RT-PCR performed on cerebrospinal fluid was negative. The second case-patient experienced an ocular disease 3 weeks after symptom onset. No hemorrhagic fever or deaths related to RVF have been reported since the outbreak onset.

Among livestock, the first confirmed case was reported on December 4, 2018. Clinical signs were reported by the veterinarian at the time of blood sampling. Among the 165 cases confirmed, 121 (100 cattle and 21 small ruminants) had aborted and 44 showed signs such as hyperthermia, nasal discharge, or digestive disorders.

After the first case in a human was diagnosed, the monitoring and managing protocol of the outbreak was shared by the regional unit of the Santé Publique France, the Agence de Santé Océan Indien, and the Centre Hospitalier de Mayotte laboratory. The objective of this protocol was to implement outbreak control actions (e.g., vector control), actively search for symptomatic humans, and conduct clinical and environmental investigations. Two weeks later, the first case in an animal was confirmed and veterinary services conducted surveys.

Public health authorities informed the general population about the situation through media and social networks to encourage persons to take preventive measures against mosquito bites. Farmers and others involved with slaughtering animals are trained to protect themselves from infectious disease agents. In addition, since February 27, 2019, selling raw and curdled milk has been prohibited.

## Conclusions

An outbreak of RVF in Mayotte resulted in 142 confirmed cases in humans as of July 31, 2019, and several clusters among livestock were confirmed. The mode of transmission is not well identified for all cases, but most commonly reported were exposure to animals or consumption of raw milk (a common practice in Mayotte). Some evidence indicates that humans may become infected with RVFV by ingesting the unpasteurized or uncooked milk of infected animals (*5*).

Our surveillance system for dengue-like syndrome is particularly sensitive and has enabled detection of the first RVF case-patients requiring hospitalization. However, given the high number of asymptomatic and paucisymptomatic forms of illness reported in the literature, the epidemiologic situation may be underestimated. The extent of the epidemic could be assessed by a seroprevalence survey at the end of this outbreak.

This epizootic occurred in the context of increased illegal imports of animals (goats, sheep, and cows) over several months from potential disease-endemic/epidemic countries. Infected animals, especially sheep, were identified among intercepted animals.

RVFV, which was identified in Kenya, is still present in eastern Africa (*14*), but little information is available about its seroprevalence on the Indian Ocean islands. Mayotte is at risk for introduction and circulation of infectious agents involved in outbreaks in neighboring countries, such as recent infections and circulation of RVFV in the Comoros Islands (*15*).
